# Corneal Opacity Induced by Antiglaucoma Agents Other Than Brimonidine Tartrate

**DOI:** 10.1155/2020/4803651

**Published:** 2020-05-26

**Authors:** Yuka Kasuya, Ichiya Sano, Shinji Makino, Hidetoshi Kawashima

**Affiliations:** Department of Ophthalmology, Jichi Medical University, Shimotsuke, Tochigi, Japan

## Abstract

The aim of this study is to report a patient with corneal opacity that developed after the use of topical antiglaucoma medications other than brimonidine tartrate (BT). An 85-year-old woman presented with corneal opacity and neovascularization in both eyes. A diagnosis of glaucoma was made 20 years previously, and antiglaucoma agents were prescribed (latanoprost, tafluprost, timolol maleate, travoprost, bimatoprost, ripasudil hydrochloride hydrate, and brinzolamide/timolol maleate) for both eyes. Ocular examination revealed semicircular fan-shaped corneal sterile infiltration with neovascularization. Anterior-segment optical coherence tomography (OCT) showed marked corneal opacity and thickened corneal stroma. The topical drugs were discontinued and replaced with 0.1% betamethasone eye drops. Two weeks after topical drugs were discontinued and replaced with betamethasone, the corneal sterile infiltration markedly improved, although the corneal opacity remained across the stromal layer. In addition, corneal opacity, intermixed with separate transparent sections, was observed as a striped shape. OCT showed an improvement of the thickened corneal stroma. Six weeks after the initial visit, the remaining corneal opacity could be seen as a mixture of opaque and nonopaque areas in stripes. The corneal stromal thickness decreased almost back to the normal range, while the area of the corneal opacity remained unchanged. *In vivo* laser confocal microscopy showed hyperreflective materials with needle-like structures in the corneal stroma. The corneal opacity showed several similarities to the previous reports of the cases treated with BT. Therefore, clinicians should be mindful of a possible development of corneal opacity in patients treated with antiglaucoma medications other than BT.

## 1. Introduction

Brimonidine tartrate (BT) ophthalmic solution, a topical *α*2-adrenoreceptor agonist, has been used for glaucoma therapy, and reports of adverse side effects include allergic conjunctivitis, blepharitis, and conjunctival hyperemia [1]. However, corneal side effects are uncommon [2]. In 2017, Maruyama et al. [3] reported two cases of peripheral corneal opacity that developed after treatment with BT. To date, four reports including eight cases have been described in Japan [3–6]. In this study, we report a case of bilateral corneal stromal opacity, which showed several similarities to previous cases treated with BT that presumably developed after treatment with topical antiglaucoma medications other than BT.

## 2. Case Presentation

An 85-year-old woman presented with corneal opacity and neovascularization in both eyes. She was diagnosed as having primary open-angle glaucoma 20 years previously and had been treated with seven different ocular hypotensive agents (latanoprost, tafluprost, timolol maleate, travoprost, bimatoprost, ripasudil hydrochloride hydrate, and brinzolamide/timolol maleate) for both eyes (OU). In detail, she was initially treated with latanoprost eye drops (Xalatan; Pfizer Inc., New York, USA), with timolol maleate (Timoptol, Santen Pharmaceutical Co. Ltd., Osaka, Japan) being added to her treatment regimen in 2005. In April 2013, her treatment was switched from latanoprost to tafluprost (Tapros, Santen Pharmaceutical Co. Ltd., Osaka, Japan). In May 2014, her treatment was switched from timolol maleate to brinzolamide/timolol maleate (Azorga Combination Ophthalmic Suspension, Novartis, Basel, Switzerland). In April 2015, her treatment was switched from tafluprost to travoprost (Travatan Z Ophthalmic Solution 0.004%, Novartis, Basel, Switzerland) and then to bimatoprost (Lumigan, Senju Pharmaceutical Co. Ltd., Osaka, Japan) in November 2017. Furthermore, ripasudil hydrochloride hydrate (Glanatec ophthalmic solution 0.4%, Kowa Co. Ltd., Nagoya, Japan) was added to her treatment regimen for both eyes since October 2016. Thus far, she was being treated with three kinds of eye drops (brinzolamide/timolol maleate, ripasudil hydrochloride hydrate, and bimatoprost) at the time of the consultation. She has had no history of BT use. Corneal opacity was observed since December 2018 at the referral eye clinic. She had no remarkable ophthalmological, medical, allergic histories and rosacea.

**Figure 1:**
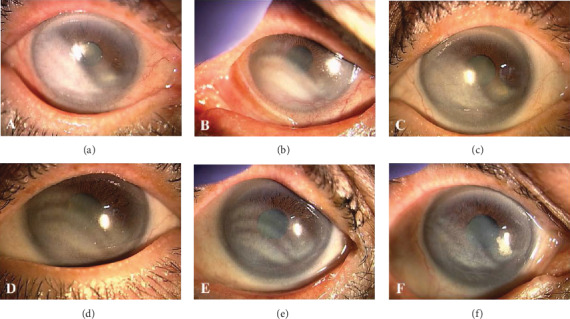
Corneal findings in the right (a, c, and e) and left (b, d, and f) eyes. (a, b) Slit-lamp examination shows corneal sterile infiltration and neovascularization in the temporal-lower side of the right eye and in the nasal-lower side of the left eye at the initial visit. (c, d) Two weeks after the initial visit (after topical agents were discontinued and replaced with betamethasone), slit-lamp examination showed a marked regression of the corneal vascularization and decreased corneal opacity bilaterally, while the area of the corneal opacity remained unchanged. (e, f) Six weeks after the initial visit, corneal opacity, intermixed with separate transparent sections, was clearly seen bilaterally, while the area of the corneal opacity remained unchanged.

**Figure 2:**
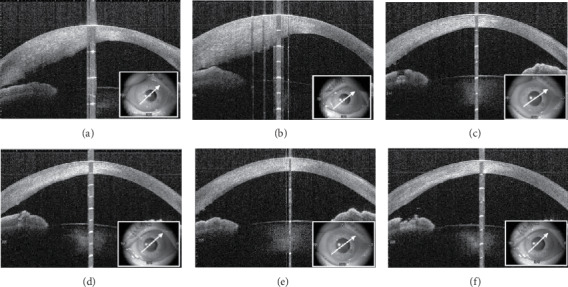
Anterior-segment optical coherence tomography (OCT) images in the right (a, c, and e) and left (b, d, and f) eyes. (a, b) Anterior OCT images showing opacity deep in the marked thickening corneal stroma in both eyes at the initial visit. (c, d) Two weeks after the initial visit (after topical agents were discontinued and replaced with betamethasone), an improvement of the thickened corneal stroma was observed. (e, f) Six weeks after the initial visit, corneal stromal thickness improved almost back to normal.

**Figure 3:**
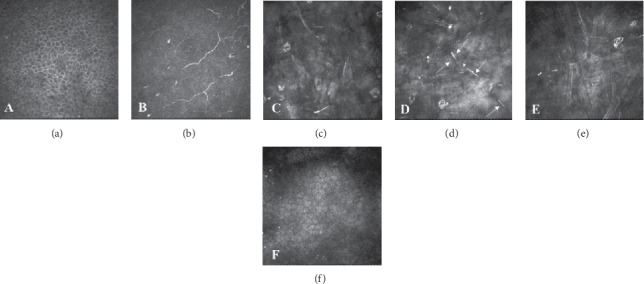
*In vivo* confocal microscopic (IVCM) images (a–f): (a) the basal epithelium (12 *μ*m depth), (b) subbasal nerve plexus (129 *μ*m depth), (c) anterior stroma (206 *μ*m depth), (d) mid stroma (235 *μ*m depth), (e) posterior stroma (266 *μ*m depth), and (F) endothelium (483 *μ*m depth). IVCM showed hyperreflective materials with needle-like structures in the corneal stroma (d arrows). Each image represents a 400 × 400 *μ*m^2^ area.

At the initial presentation, the best-corrected visual acuities (BCVAs) were 0.3 in her right eye (OD) and 0.15 in her left eye (OS) in decimal; the intraocular pressures (IOPs) were 11 mmHg OD and 15 mmHg OS. Bilateral gonioscopy showed open iridocorneal angles, and fundoscopy showed typical glaucomatous changes in the optic nerve heads OU. Ocular examination revealed follicular conjunctivitis and blepharitis OU and corneal sterile infiltration with neovascularization in the temporal lower side OD and in the nasal lower side OS (Figures 1(a) and 1(b)). Anterior-segment optical coherence tomography (OCT) showed a marked corneal opacity and thickened corneal stroma OU (Figures 2(a) and 2(b)). Although she had no history of BT use, she was diagnosed with stromal opacity with deep corneal vascularization possibly associated with certain topical medications. Therefore, we discontinued all three ocular hypotensive agents and initiated treatment with 0.1% betamethasone sodium phosphate (Sanbetason ophthalmic and otorhinologic solution 0.1%, Santen Pharmaceutical Co. Ltd., Osaka, Japan) four times daily OU. Two weeks after the discontinuation of topical drugs and administration of betamethasone, both the blepharitis and corneal sterile infiltration markedly improved (Figures 1(c) and 1(d)). In addition, corneal opacity, intermixed with separate transparent sections, was observed as a striped shape. OCT showed an improvement in the thickened corneal stroma (Figures 2(c) and 2(d)). The BCVAs were 0.5 OD and 0.2 OS, and the IOP was 15 mmHg OU. Six weeks after the initial visit, corneal opacity, intermixed with separate transparent sections, was clearly seen bilaterally (Figures 1(e) and 1(f)). Corneal stromal thickness improved almost normally, while the area of the corneal opacity remained unchanged (Figures 2(e) and 2(f)). The BCVAs were 0.7 OD and 0.6 OS, and the IOP was 16 mmHg OU. *In vivo* laser confocal microscopy (IVCM; Heidelberg Retina Tomograph II Rostock Cornea Module) showed hyperreflective materials with needle-like structures in the corneal stroma (Figures 3(a)–3(f)). The progression of corneal thinning and definite lipid deposition was not detected during the 18-month follow-up period.

## 3. Discussion

In this study, we report a case of bilateral corneal stromal opacity that resembles previous cases that were treated with BT and that presumably developed after the use of topical antiglaucoma medications other than BT.

BT has been associated with ocular side effects including conjunctival injection, allergic conjunctivitis, and blepharitis; however, corneal side effects are uncommon [1, 2]. To date, four reports including eight cases with severe corneal disorder have been reported in Japan [3–6]. According to these reports, the clinical features of BT-induced corneal opacity were as follows. First, peripheral corneal sterile infiltration was observed, especially below the cornea, and was semicircular or fan-shaped with blepharitis. Second, corneal lesions were present deep in the corneal stroma with vascular invasion. Third, corneal disorder developed several years after the induction of topical BT. Fourth, the use of topical steroid and discontinuance of topical BT led to improved clinical symptoms. Last, the remaining corneal opacity could be seen as a mixture of opaque and nonopaque areas in stripes.

According to previous cases that were treated with BT, corneal opacity might be due to an allergic reaction, interaction between BT and other antiglaucoma medications, effects of preservatives such as benzalkonium chloride and sodium chlorite, and racial differences. However, the exact mechanism for corneal opacity remains unknown.

Additionally, lipid keratopathy can result from corneal sterile infiltration with neovascularization and can be secondary to stromal corneal herpes, other corneal diseases, trauma, contact lens wear, or systemic diseases [7–10]. In cases of lipid keratopathy, crystalline- or needle-like structures have been observed by IVCM [10]. These findings are consistent with the histopathological description, which states that the opacity of the cornea was caused by deposition of cholesterol in the corneal stroma [7]. IVCM findings in our case of corneal opacity that developed after treatment with topical antiglaucoma agents indicate the mechanisms behind; i.e., long-term use of multiple topical antiglaucoma agents led to the corneal sterile infiltration with neovascularization. As a result, serum lipid components accumulated in the corneal stroma as observed in cases of lipid keratopathy. From this point of view, IVCM can be a useful examination for the diagnosis of this rare corneal disorder in future cases.

We present a case of bilateral corneal stromal opacity, which showed several similarities to previous cases that were treated with BT and that presumably developed after treatment with topical antiglaucoma medications other than BT. The present case indicates that clinicians should therefore be mindful of a possible development of corneal opacity in patients treated with antiglaucoma medications other than BT. A further accumulation of reports may be needed.
